# Effectiveness of dental bleaching in depth after using different bleaching agents

**DOI:** 10.4317/jced.51063

**Published:** 2013-04-01

**Authors:** Maria B F. D’Arce, Débora A N L. Lima, Flávio H B. Aguiar, Carlos E S. Bertoldo, Gláucia M B. Ambrosano, José R. Lovadino

**Affiliations:** 1 DDS, MS, PhD Student. Department of Restorative Dentistry, Piracicaba Dental School, University of Campinas-UNICAMP, Piracicaba, SP, Brazil; 2 DDS, MS, PhD, Assistant Professor. Department of Restorative Dentistry, Piracicaba Dental School, University of Campinas-UNICAMP, Piracicaba, SP, Brazil; 3 PhD, Assistant Professor. Department of Social Dentistry/Statistics, Piracicaba Dental School, University of Campinas-UNICAMP, Piracicaba, SP, Brazil; 4 DDS, MS, PhD, Full Professor. Department of Restorative Dentistry, Piracicaba Dental School, University of Campinas-UNICAMP, Piracicaba, SP, Brazil

## Abstract

Objectives: This study evaluated the effectiveness of low- and high-concentration bleaching agents on enamel and deep dentin. Study design: Stained bovine incisors fragments were randomized placed into 10 groups (n=5), according to the sample thicknesses (2.0 mm or 3.5 mm) and bleaching agent: 10% carbamide peroxide (CP) (4 h a day/21 days); 6% hydrogen peroxide (HP) with calcium (1:30 h a day/21 days); HP 20% with calcium (50 min a day/3 sessions with a 7-day interval); HP 35% (3 x 15 min a day/3 sessions with a 7-day interval); HP 35% with calcium (40 min a day/3 sessions with a 7-day interval). The samples were stored in artificial saliva during the experiment. The color change was evaluated using a spectrophotometer at the initial analysis, after artificially staining with black tea and after each of the bleaching weeks, and data was expressed in CIE Lab System values. The L* coordinate data was submitted to analysis of variance and Tukey-Kramer test and the ?E values data was submitted for analysis of variance in a split-plot ANOVA and Tukey’s test (?=0.05). Results: None of the bleaching agents tested differed from the reflectance values on the enamel surface. For deep dentin HP 20% and HP 35%, both with calcium, showed the lowest reflectance values, which differed from CP 10%. Conclusion: It is concluded that high concentration hydrogen peroxide with calcium was less effective in deep dentin than 10% carbamide peroxide.

** Key words:**Dental bleaching; hydrogen peroxide; carbamide peroxide; dental staining.

## Introduction

Several methods and approaches have been described in the literature for bleaching vital teeth, such as the use of different bleaching agents, concentrations, times of application and product format (1). The commonly used bleaching agents are hydrogen and carbamide peroxide in several concentrations. Haywood and Heymann (2) presented the technique of “night-guard vital bleaching,” that offers a safe and effective way of bleaching mildly discolored teeth using a soft custom tray with 10% carbamide peroxide worn by the patient at night. The use of a high concentration of bleaching agents, called “in-office” bleaching, seems to be an appropriate alternative to home bleaching applications (3) since the treatment is performed in an office with weekly sessions and then the product application time is less compared with home bleaching.

The mechanism by which teeth are whitened by oxidizing materials such as hydrogen peroxide and carbamide peroxide are not fully understood (4). The dissociation of hydrogen peroxide into free radicals may be influenced by the temperature, pH, and co-catalysts, leading to generation of different types of oxygen more or less potent than the original molecule (5). Also, the permeability of hydrogen peroxide through the structure of enamel and dentin depends of the concentration of the product as well as its exposure time on enamel surface (6).

Several studies have evaluated the different concentrations of hydrogen and carbamide peroxides on bleaching efficacy over the years (7-9). According to Sueliman et al. (10), there was a relationship between the number of applications and the concentration of bleaching gels and the higher concentration of hydrogen peroxide needed a lower number of applications to achieve the same efficiency of whitening. Bernardon et al. (11) reported a similarity between the in-office and home bleaching techniques. A number of studies have reported the effectiveness of bleaching agents, but only a few paid direct attention to both in-office and at home approaches (12,13), simulating the clinical intraoral situation and assessing the color changes on the enamel and dentin surfaces. The gel concentration increase allows a reduction in the time application of the bleaching agent, since a greater amount of active ingredient will be available (14).

Therefore, in-office bleaching has many advantages compared with home treatments, such as a short agent bleaching exposition, no need to use trays, total control of the procedure performed by a dentist who does not depend on patient compliance, and others (15). However, it is unclear if the hydrogen and carbamide peroxides’ diffusion into dental structures allows a similar color change in deep dentin. According Garber et al. (16), office treatments without association with home bleaching achieve lower color stability. Thus, the purpose of this study was to evaluate the bleaching treatment efficacy on color changes of enamel and deep dentin after bleaching with high- and low-concentration agents containing calcium or not in their composition.

## Material and Methods

Fifty bovine incisors were stored in a thymol solution at 0.1% after collection and disinfection. These teeth were examined under a light microscope (4x) to investigate for the presence of gaps, cracks or any kind of pigmentation that would interfere with the bleaching evaluation. If some of these features were found, the tooth was discarded and replaced.

The crowns were separated from their roots through a section in the dentin-enamel junction using a double-faced diamond disc (KG Sorensen, Barueri, SP, Brazil) in a low-speed hand piece under constant water irrigation. The crowns were cut with a precision, slow-speed, water-cooled diamond saw (Imptech PC10, EquilamLab Equip., Diadema-SP, Brazil, 09960-500) to obtain blocks with an area of 25 mm2 (5mm wide x 5mm long).

The enamel surface was flattened with silicon carbide (SiC) paper of #600 and #1200 grit under constant irrigation. Likewise, the dentine surface was abraded with SiC #600 and #1200 grit, achieving a block 2.0-mm thick (1 mm of enamel and 1 mm of dentin) or 3.5 mm thick (1.0 mm of enamel and 2.5 mm of dentin). Each specimen was marked with a diamond bur #1012 (KG Sorensen, Barueri, SP, Brazil) on one of the sides to standardize the sample position in the spectrophotometer (Konica Minolta CM 700d, Japan). The bovine incisors fragments were randomized and placed into 10 groups (n=5), according to the sample thicknesses (2.0 mm or 3.5 mm) and bleaching agent protocol.

The initial reading (baseline) was performed using a spectrophotometer (CM-700d, Konica Minolta, Japan). The samples were positioned in a sample carrier to obtain the enamel and opposite dentin initial readings, taken in a light cabin (GTI Mini Matcher MM1e, GTI Graphic Technology Inc., Newburgh, NY, USA) to standardize the ambient light during the measurement process, and then the samples were subjected to a reading with the spectrophotometer. The test measures L*, a*, and b* color space and this system are referred to as CIEL*a*b*. In the color space, L* indicates lightness (L + = lightness and L - = darkness), the a* coordinate represents the red/green range (a* + = redness and a* - = greenness) and the b* coordinate represents for the yellow/blue range (b* + = yellowness and b* - = blueness). The values of the coordinates a* and b* approach zero, indicating neutral colors (white and gray) and an increase in magnitude for more saturated or intense colors (17). The L*a*b* system allows the numeric definition of a color as well as the difference between two colors using the following formula: ?E= [(L1 – L0)2 + (a1 – a0)2 + (b1 – b0)2]1/2. The data was read by a microcomputer using On Color QC Lite software (Konica Minolta, Japan) to generate spectral measurements as a function of wavelength for data processing and analysis.

After the baseline reading (Time 1), the dentin of each block was protected with sticky wax, and then the sample was immersed in a solution of black tea for six days at room temperature in order to stain the enamel surface (18) and adjacent dentin by diffusion. The tea solution was obtained by soaking 1.6 g of tea (black tea: Leão) in 100 ml boiling water for five minutes. The solution was changed every 24 hours for six days. The samples were stored in artificial saliva for 15 days to stabilize the staining and then a new color measurement was performed (Time 2) (19). Before this reading, superficial enamel was cleaned with pumice, using a polishing rubber mounted at a contra angle to remove extrinsic stains.

For bleaching procedures, dental blocks were fixed in a device and approximately 1.0 mm of the bleaching agent was applied on the enamel surface. The gel was applied according to the manufacturer’s instructions. The bleaching procedures were performed according to the following protocols:

Groups 1 (2.0 mm) and 2 (3.5 mm): 10% carbamide peroxide (Whiteness Perfect – FGM, Santa Catarina, Brazil): The bleaching agent remained in contact with the enamel surfaces for 4 h. The samples were inserted into an apparatus containing water to avoid dehydration, but without water contact with the surface containing the gel. Bleaching was performed daily for 21 days.

Groups 3 (2.0 mm) and 4 (3.5 mm): 6% hydrogen peroxide with calcium (White Class – FGM, Santa Catarina, Brazil): The bleaching agent was applied in a similar manner according to groups 1 and 2; however, the gel remained on the enamel surface for 1:30 hr. A daily session of bleaching was performed for 21 days.

Groups 5 (2.0 mm) and 6 (3.5 mm): 20% hydrogen peroxide with calcium (Whiteness HP Blue–FGM, Santa Catarina, Brazil): The bleaching agent was applied on the enamel surface for 50 min. Three bleaching sessions were performed and the interval between sessions was seven days.

Groups 7 (2.0 mm) and 8 (3.5 mm): 35% hydrogen peroxide (Whiteness HP Maxx–FGM, Santa Catarina, Brazil): In every application, the bleaching agent was applied on the enamel surfaces for 15 min. Three gel applications were made in each session. Three bleaching sessions were performed and the interval between sessions was seven days.

Groups 9 (2.0 mm) and 10 (3.5 mm): 35% hydrogen peroxide with calcium (Whiteness HP Blue–FGM, Santa Catarina, Brazil): The bleaching agent was applied on the enamel surface for 40 min. Three bleaching sessions were performed and the interval between sessions was seven days.

The entire procedure was performed in a temperature-controlled environment (23.0 ± 1°C). At the end of each bleaching session, samples were thoroughly washed in running water, dried with absorbent paper and stored in artificial saliva at a temperature of 37°C ± 2. At the end of each bleaching week, there was a waiting period of 24 h for the specimens to rehydrate before spectrometer readings were taken (Times 3, 4 and 5).

For L* values, the specimens’ “sample size” was calculated using SAS Power and Sample Size program reached 40 “error freedom degree” for the main effects (treatment, thickness, and surface) and their interactions in these treatment designs. For time and its interactions with other factors, the “freedom degree” was 322, which was considered very high for variability around 1.25%. This provided a significance level of 5% in a power test with a result above 0.90 for main effects and interactions. For ?E values, the “freedom degree” was also 40 for treatment, thickness, and surface, and their interactions were also considered a high value for this variability, providing up to a 5% significance level and the power of the test was above 0.80 for all main effects and interactions.

After exploratory data analysis, the variable L* was analyzed, considering repeated measures by the MIXED procedure of SAS. For the choice of the matrix of variance and covariance, Akaike information criterion was used, selecting the one with the lowest value for this parameter. The adjusted means were obtained through the LSMEANS and the mean comparison was performed using the Tukey-Kramer test at a significance level of 5%. The variable ?E was analyzed, considering repeated measures by the MIXED procedure of SAS and Split-plot ANOVA and Tukey’s Test at a significance level of 5%.

## Results

- L* Values (L=100 – lightness; L=0 – darkness)

**Figure 1:**
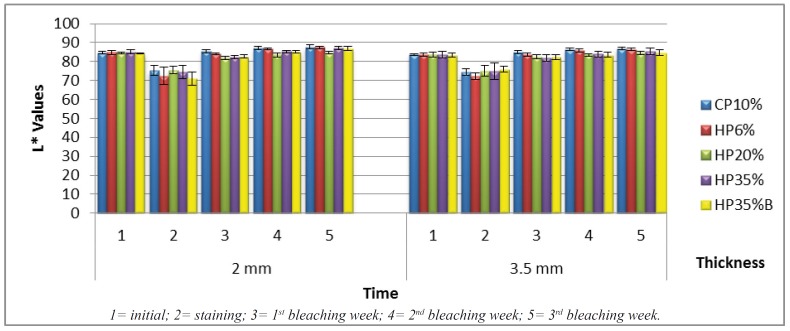
Enamel L* means (SD) in function of treatment and time.

Data shown in Fig. 1 (L* enamel values) that the treatments were not statistically different for the enamel surface in two evaluated thickness during the bleaching process (Times 3, 4 and 5).

**Figure 2:**
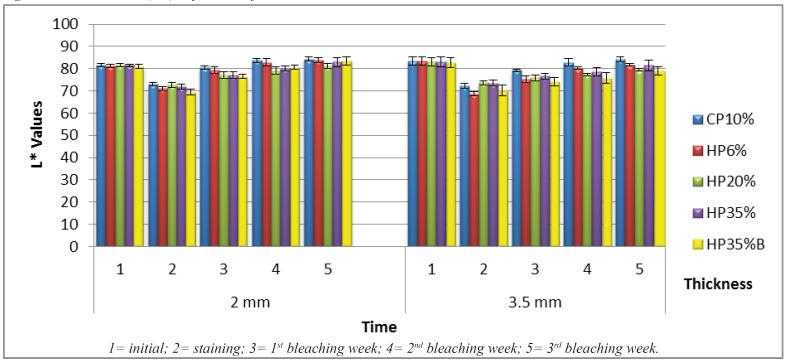
Dentin L* means (SD) in function of treatment and time.

In Fig. 2 (L* dentin values) for samples with a thickness of 3.5 mm, CP10% showed the highest L* value, dif-fering from HP20% and HP35%B in Times 4 and 5 on the dentine surface.

- Delta E (?E)

 Table 1 refers the color variation media (?E1) between the bleaching session (time) Black Tea Stain x First Session Bleaching. For samples 2-mm thick, the enamel surface showed a higher ?E1 for CP 10%, HP 6% and HP 35% with calcium and was statistically different from HP 20% with calcium. For 3.5-mm thick samples, CP10% and HP6% showed a higher ?E1 compared to HP 35% and HP 35% with calcium on the enamel surface.

**Table 1:**
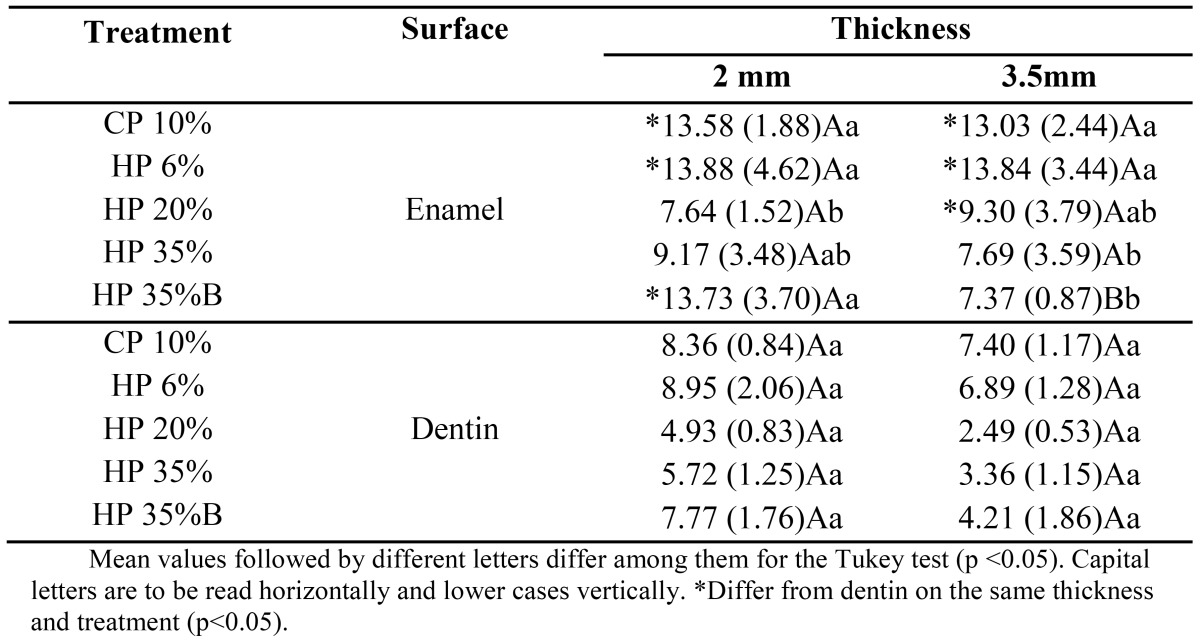
?E1 (Staining x 1st Bleaching week.

 Table 2 presents the ?E2 media for “First Bleaching Week x Second Bleaching Week.” On the dentin surface, CP 10% and HP 6% had the highest ?E2, differing from the other treatments for samples with a 3.5 mm thick-ness.

**Table 2:**
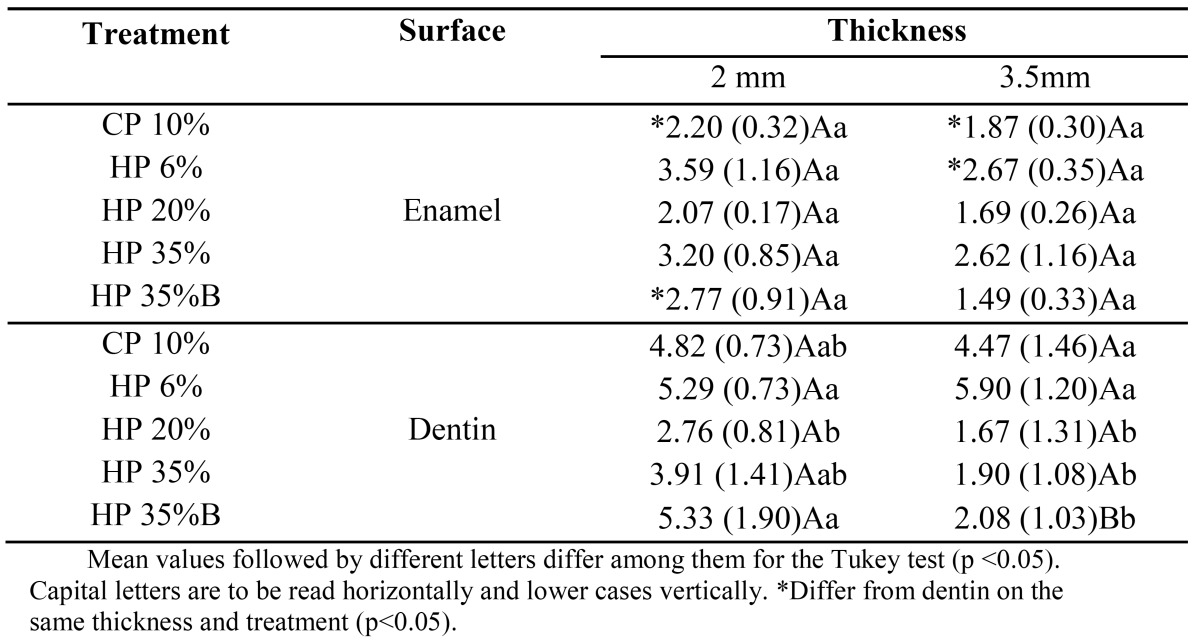
?E2 (1st Bleaching week x 2nd Bleaching week

 Table 3 presents the ?E3 media of color variations between the second and third week of the bleaching process. For dentin of 3.5-mm thick samples, there was no significant difference between treatments.

**Table 3:**
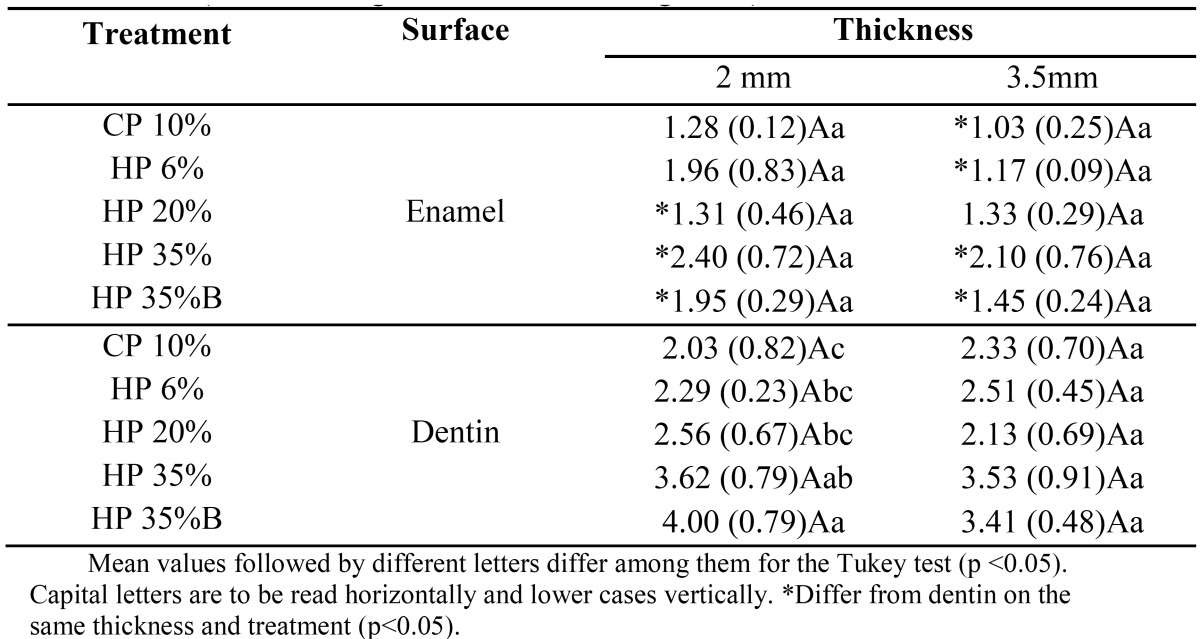
?E3 (2nd Bleaching week x 3rd Bleaching week

For HP 35% with calcium, the 2.0-mm thick samples statistically differed from the 3.5-mm thick ones on the enamel surface, presenting a higher ?E1, and on the dentin surface, presenting a higher ?E2.

## Discussion

The use of bovine teeth is justified because they allowed the preparation of specimens with standardized-sized enamel and dentin all along them, since the bovine incisors have a flat buccal surface (12,20). According to Hanning et al. (21), the physical-chemical characteristics of bovine teeth do not differ considerably from human dentin. The diameter of the tubule of bovine dentin is 3-5µm with 20.000 tubules/mm2 and human dentin, in the outer layers near enamel, is 0.5-1.2 µm with 10.000 -25.000 tubules/mm2. This is an important factor when the peroxide diffusion is quantified (21). However, this was not evaluated in the present study. The aim of this study was to evaluate the color change of subjacent dentin with a similar experimental condition for all groups. Attia et al. (22) concluded in an in vitro study that bovine and human dental substrates behave similarly during the bleaching process, probably due the similarity of morphological.

Color differences in bovine dental fragments were evaluated through reflectance measurements with the CIE Lab Color coordinate system. According to Dietschi et al. (20), when the three coordinates of color dimensions are analyzed separately, L* values, which depict the object lightness, appeared to be the most relevant parameter to make comparisons under experimental conditions.

The treatments studied did not differ for L* values on the enamel surface. Nevertheless, the dentin surface of the 3.5 mm samples treated with HP 20% with calcium and HP 35% with calcium showed less whitening effects than the CP 10%. These findings are in agreement with Dietsch et al. (12) who found that high-concentrate bleaching gels were less efficient than low-concentration ones for removing stains deposited in dentin. On the other hand, Matis et al.(23) reported that the low-concentration bleaching agents used in the overnight system for six to eight hours a day had better outcomes compared with high-concentration bleaching treatments. The 10% carbamide peroxide treatment has proven its effectiveness over the years, since the technique was described by Haywood and Haymman in 1989 (2).

According to Sun (5), the carbamide peroxide dissociates into hydrogen peroxide and urea. Then, the urea continues to decompose into CO2 e ammonia, considered a strong base that raises the pH environment and allows a higher perhydroxil (HOO-) production. This is the strongest free radical formed from hydrogen peroxide ionization and reacts with the organic pigments during the bleaching process. In addition, the CP 10% contains a lower concentration of hydrogen peroxide available to bleaching reaction compared to the others bleaching agents in the present study, therefore it needs a longer time exposure in contact with the tooth surface to achieve a satisfactory bleaching result. According to Marshall et al. (24), the carbamide peroxide presents bleaching effectiveness even after six hours using the gel in trays, so their longer half-life justifies their best performance.

According to Kwon (25), hydrogen peroxide apparently has the power to produce microporosity on the tooth surface while degrading the organic material during bleaching. This phenomenon would occur, regardless of the gel’s pH and despite these changes not being clinically perceived. They occur mainly through the use of a high-concentration bleaching agent. Lately, small amounts of calcium have been added to high-concentration bleaching gels, since the calcium present may decrease the speed of mineral loss reactions (26). In the present study, the dentin surface of 3.5-mm samples did not reach the initial L* values (before staining) after a bleaching treatment with HP 20% and HP 35%, both with calcium. The hypothesis suggested is that calcium gluconate in these gels may have interfered with the transitional mineral exchanges that occurred during the bleaching process, resulting in a worse action of the gels. The bleaching agents may be more calcium saturated momentarily than the enamel surface, and therefore the calcium ionic released from the gel to the substrate (27) may make the transitional microporosity formation more difficult, leading to less effective bleaching in deeper portions of the tooth.

This study also evaluated ?E, which shows the magnitude of the change and not the direction of the change in the three coordinates of the CIELab System (28). The ?E1 values ( Table 1), comparing the color changes of the samples at time of “Staining x the Bleaching 1st week,” showed generally higher values in the enamel than on the dentin surface for all treatments studied. The contact of the bleaching gel directly on the superficial pigmentation may have allowed better removal of this stain on the enamel surface. As the bleaching gel remained in contact with the enamel surface, it is expected that oxidation of the pigments by peroxide on this surface is more effective at first, before bleaching the deeper dentin.

The greatest variations of ?E1 occurred on enamel surface of 3.5 mm samples when low-concentration agents CP 10% and HP 6% were used, compared with HP 35%. On the other hand, the ?E2 (“Bleaching 1st week x Bleaching 2nd week”) CP10% and HP 6% statistically differed from high-concentration bleaching agents for dentin surfaces, reversing the first condition (?E1). These results show that the bleaching agent acts more efficiently in deep dentin after the removal of the superficial staining of the enamel. For ?E3 (“Bleaching 2nd week x Bleaching 3rd week”), the treatments did not differ, demonstrating a non-significant color change between these two sessions.

The CP 10% had the best performance on the dentin surface of the 3.5 mm thick samples and obtained the best results in comparison with gels of high concentration with calcium. These findings are in agreement to Zekonis et al. (29) that compared, in vivo, the efficacy of low- and high-concentration bleaching agents, achieving the best results for home bleaching treatments. In the same way, Dietschi et al. (20) evaluated the efficacy of various bleaching treatments on enamel and dentin of bovine dental blocks pigmented with blood. The authors reported that all products had a similar bleaching effect on the enamel surface, while for dentin the best bleaching results were found in low concentration peroxides.

Sulieman et al. (30) evaluated the efficacy of hydrogen peroxide gels with concentrations ranging from 5% to 35% and concluded that the higher concentration of the product would need fewer applications for a satisfactory bleaching. This statement is not in agreement with results of the present study, since the high-concentration bleaching agents needed one more session to reach their best results compared with low-concentration gels in the deepest dentin.

Also, the thickness of dentin can negatively interferes on color changes of enamel. The HP35% with calcium showed lower bleaching on enamel surface in 3.5 mm thickness samples compared to 2mm. Although the gel remains in contact with the both enamel surface for the same time, the color reflected from a larger portion of stain dentin through enamel translucency probably caused this difference. According to Joiner (17), light scat-tering and absorption within dental hard tissues give rise to the intrinsic color of the teeth and as the enamel is a relatively translucent surface, the properties of dentine are very important to determining the overall tooth color.

Efforts have been made in order to achieve the maximum efficacy of bleaching agents in color changes of teeth in less time and with minimal side effects. This attempt to minimize the transient mineral loss of enamel by adding calcium to high-concentration bleaching agents may adversely affect the whitening action of gel in deep dentin, thus making the color change of tooth more superficial. Perhaps this may accelerate the rebounding whitening effect. In vivo studies are needed to confirm these findings.

## Conclusion

The present study demonstrated that the whitening efficacy on the enamel surface did not depend on the bleaching agents concentrations. However, the high-concentration hydrogen peroxide with calcium was less effective in deep dentin than the 10% carbamide peroxide.
